# Regular transient limb ischemia improves endothelial function and inhibits endothelial cell apoptosis to prevent atherosclerosis in rabbit

**DOI:** 10.1186/s12872-024-03869-0

**Published:** 2024-04-16

**Authors:** Nan-rong Zhang, Yi Wen, Jing Li, Wan-jun Zheng, San-qing Jin

**Affiliations:** 1 Department of Anesthesia, The Sixth Affiliated Hospital, Sun Yat-Sen University, Guangzhou, 510655 Guangdong China; 2 Biomedical Innovation Center, The Sixth Affiliated Hospital, Sun Yat-sen University, Guangzhou, 510005 Guangdong China; 3 Department of Anesthesia, Guangzhou Women and Children’s Medical Center, Guangzhou, 510623 Guangdong China

**Keywords:** Regular transient limb ischemia, Atherosclerosis, Endothelial, Apoptosis

## Abstract

**Aims:**

Regular transient limb ischemia (RTLI) can prevent atherosclerosis (AS) progression in hypercholesterolemic rabbits. This study aimed to investigate the minimum effective intensity and possible mechanisms of RTLI for preventing atherosclerosis.

**Methods:**

Eighty rabbits were divided into eight groups: normal (N), high cholesterol (H), three RTLI [three RTLI cycles every other day (R3qod), three RTLI cycles daily (R3qd), and six RTLI cycles daily (R6qd), each cycle of RTLI included 5 min of limb ischemia followed by 5 min limb reperfusion], and three correlated sham RTLI [sham ischemia for 30 min once every other day (S3qod), sham ischemia for 30 min once daily (S3qd), and sham ischemia for 60 min once daily (S6qd)]. Rabbits in group N were kept normally, while the others were fed 1% cholesterol diet for 12 weeks. The RTLI and sham RTLI groups were received RTLI or sham RTLI procedure, respectively. The plaque area in the thoracic aorta was determined by oil red O staining, and quantifying the ratio of plaque area to intimal area (PA/IA). Endothelium-dependent and -independent relaxation were also determined. Endothelial cell were isolated from abdominal aorta of rabbits, and the apoptosis ratio was detected using flow cytometry.

**Results:**

The PA/IA and early apoptotic cell ratio was significantly lower as well as the endothelium-dependent relaxation response was higher in group R6qd than those in groups H and S6qd, while those in the R3qod group was not significantly different from those in groups H and S3qod, as well as those in the R3qd group showed no significant difference compared to those in groups H and S3qd.

**Conclusions:**

Six cycles of RTLI daily was the optimal effective intensity to prevent AS progression in rabbits. Endothelial function improvement and apoptosis inhibition might contribute to the anti-AS effects.

## Introduction

Atherosclerosis (AS) is the primary cause of morbidity and mortality in patients with cardiovascular diseases [1]. Lipid-lowering drugs are the main therapy, but interventional or surgical therapy is often applied. Nonetheless, the morbidity and mortality associated with atherosclerotic cardiovascular disease remain high worldwide. Furthermore, clinical treatment for AS remains difficult since there is no recognized method to prevent endothelial dysfunction or improve the entire vascular bed [2]. Therefore, simpler and more effective treatments must be developed. Endothelial dysfunction is accepted as an initiator and key factor in the development, progression, and clinical complications of AS [3, 4]. Therapy-induced improvement of endothelial function is related to the reduction in further atherosclerotic events [5, 6]. Therefore, protecting the endothelial function is an important target in the prevention and treatment of AS and its related diseases [4]. A single application of transient limb ischemia (TLI) was introduced as a noninvasive and effective stimulus to induce remote ischemic preconditioning by Kharbanda et al [7]. These authors reported that three 5 min cycles of transient ischemia of the forearm induced by inflating a blood pressure cuff to 200 mmHg provided potent protection against ischemia–reperfusion-induced endothelial dysfunction in the contralateral arm [7]. Subsequent studies have shown that repetitive or long-term TLI performed once or twice daily over a week [8, 9] or a month [10] also has protects endothelial functions. Furthermore, this potent endothelial protection was sustained 8 days after the cessation of repetitive TLI [8], which was longer lasting than the “late phase” protection of single-application TLI [11]. Whether this repetitive dose of TLI has lasting protective effects on the chronic progressive vascular disease AS remains unknown.

In 2009, it was hypothesized that regular TLI (RTLI) prevents AS progression by protecting endothelial cell function. An experiment was then conducted in which six cycles of TLI (5 min limb ischemia and 5 min reperfusion) were administered daily to high-cholesterol diet fed rabbits for 12 weeks. The results showed that RTLI could reduce the proportion of plaque area in the aorta, which demonstrated that six cycles of RTLI over a span of 12 weeks could prevent AS progression [12]. Furthermore, the same cycle of RTLI could protect endothelial cells by improving the vasodilation function [13]. However, whether lower intensity RTLI would have similar preventive effects on AS remains unknown, as do the possible mechanisms.

Recently, researchers have attempted to translate the beneficial effects of long-term TLI on endothelial function into clinical settings, making it an attractive strategy against endothelial dysfunction-associated diseases. To the best of our knowledge, few studies have focused on the application of repetitive or long-term TLI for the prevention and/or treatment of AS-related diseases. Meng et al. reported that brief repetitive bilateral arm ischemic preconditioning performed twice daily for 300 days was able to reduce recurrent strokes and improve cerebral perfusion status in patients with intracranial arterial stenosis [14]. Liang et al. reported that regular remote ischemic preconditioning repeated three times daily for 20 days improved endothelial function through flow-mediated dilation in patients with coronary heart disease [15]. However, Meng’s and Liang’s studies performed repetitive remote ischemic preconditioning in subjects with AS-related diseases and focused on neither the direct effects of RTLI on AS nor its possible mechanisms. Our study simulated the formation of AS by feeding a high-cholesterol diet to normal rabbits while performing daily RTLI. Additionally, our previous study confirmed the preventive effects of RTLI directly on the formation and progression of AS [12], which might partly explain the mechanism of Meng and coworker’s results. However, even though our previous study reported that endothelial protection might be one important mechanism of RTLI, further mechanisms remain unknown. The aims of the present study were first to evaluate the effects of different intensities of RTLI on AS and determine the minimum effective intensity of RTLI for AS prevention, and then second to investigate its potential mechanisms targeting endothelial cells.

## Materials and methods

### Animals and grouping

**Fig. 1:**
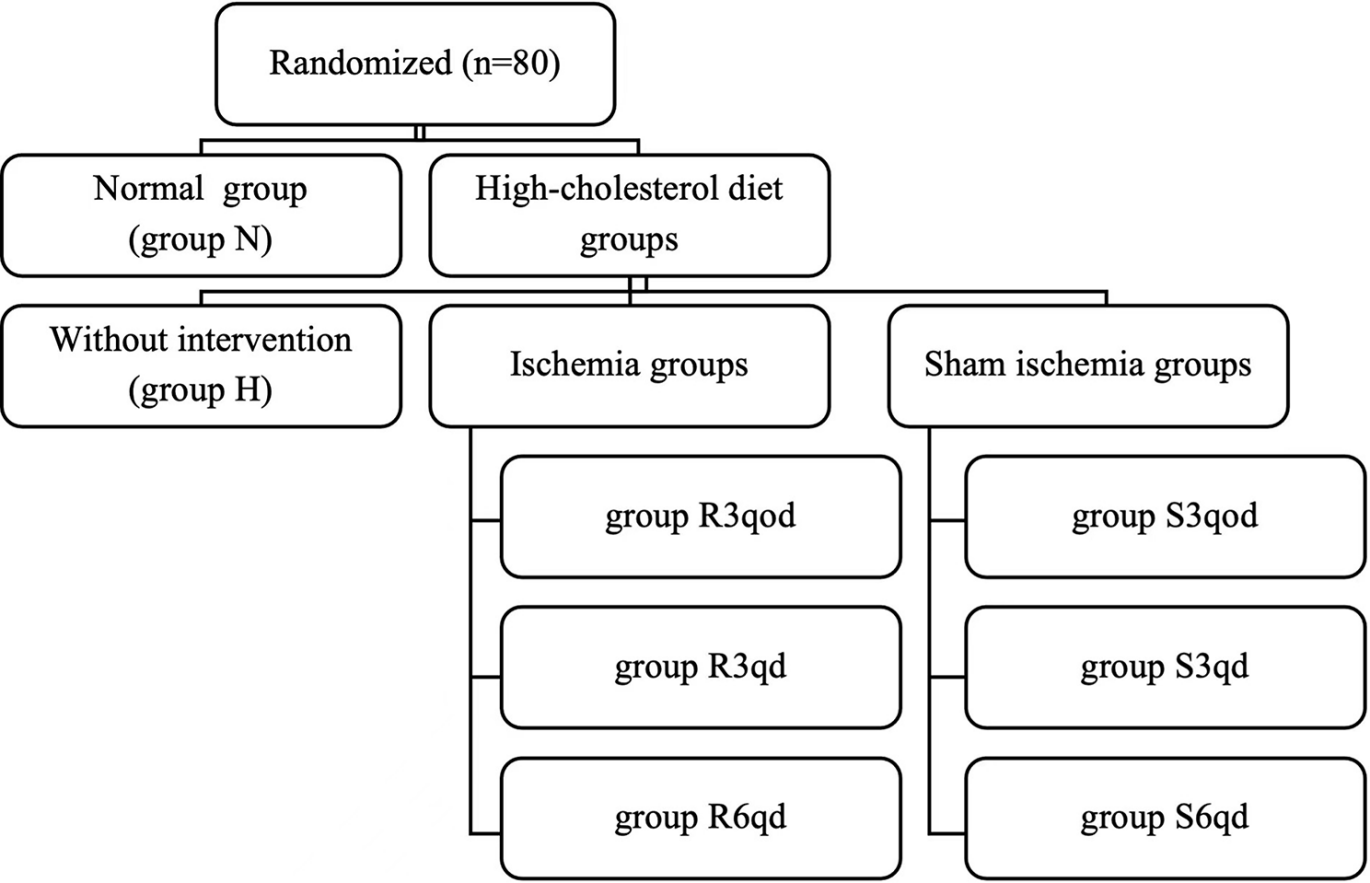
Diagram of the experimental grouping. Rabbits in group N were fed only ordinary fodder without intervention. Rabbits in group H were fed only high-cholesterol fodder without intervention. Rabbits in the R3qod, R3qd, and R6qd groups were fed with high-cholesterol fodder and received three cycles of RTLI once every other day, three cycles of RTLI once daily, and six cycles of RTLI once daily, respectively. Each cycle of RTLI included 5 min of limb ischemia followed by 5 min limb reperfusion. Rabbits in the S3qod, S3qd, and S6qd groups were fed with high-cholesterol fodder and received sham ischemia for 30 min once every other day, 30 min once daily, and 60 min once daily, respectively. RTLI, regular transient limb ischemia. *n* = 10 per group

The study protocol conformed to the Regulations for the Administration of Affairs Concerning Experimental Animals and had been approved by the Institutional Animal Care and Use Committee of Sun Yat-Sen University (No. LAEC-2013-0603). After one week of adaptation, 80 male New Zealand white rabbits weighing 2.0–3.0 kg were randomly divided into eight groups (*n* = 10 per group): normal (N), high cholesterol (H), three different intensities of RTLI groups [six cycles of RTLI once daily (R6qd), three cycles of RTLI once daily (R3qd), and three cycles of RTLI once every other day (R3qod), each cycle of RTLI included 5 min of limb ischemia followed by 5 min limb reperfusion], and three correlated sham RTLI groups [sham ischemia for 60 min once daily (S6qd), sham ischemia for 30 min once daily (S3qd), and sham ischemia for 30 min once every other day (S3qod)] (Fig. 1). The N group was fed a normal diet, while the other groups were fed a 1% cholesterol diet for 12 weeks as described previously [16]. A total of 150 g food was available for all the animals daily; food intake was recorded daily, and body weight was measured weekly.

### RTLI administration

The rabbits in the three ischemia groups were kept in rabbit hutches and received three intensities of RTLI. RTLI were achieved by a blood pressure cuff placed on the left hind limb. Each cycle of RTLI was conducted with 5 min ischemia administered by inflating blood pressure cuff to 200 mmHg, followed by 5 min reperfusion with the cuff deflated. These RTLI procedures were conducted at the beginning of high-cholesterol diet feeding, lasting for 12 weeks. The rabbits in the sham ischemia groups received the same RTLI procedures by a deflated cuff without any pressure.

All the RTLI and sham ischemia procedures were performed without anesthesia, and the rabbits were placed in a prone position and calmed by caressing. The reliability of this RTLI procedure has been demonstrated in conscious rabbits in a previous study [17]. The rabbits in groups N and H received neither RTLI nor the corresponding sham RTLI procedures.

### Serum lipid determination

At the beginning of the experiment (week 0) and the end of the 6th (week 6) and 12th weeks (week 12), blood was collected from the central ear artery the morning following a 14-h fast. Serum samples were separated by centrifugation at 4 °C, 1500 × *g* for 15 min and stored at -80 °C until analysis. Serum concentrations of total cholesterol (TC), low-density lipoprotein cholesterol (LDL-C) and high-density lipoprotein cholesterol (HDL-C) were determined using an autoanalyzer (ADVIA-2400, Bayer, Germany) [17].

### Tissue preparation

Rabbits were anesthetized by administering sodium pentobarbital (30 mg/kg) intravenously at the end of the 12th week. The left common carotid artery was carefully dissected after ligation and immediately rinsed in freshly prepared 37 °C Krebs buffer for isometric tension recording. The abdominal segment of the aorta was separated by a reference diaphragm and iliac artery bifurcation and immediately rinsed in heparin solution for the apoptotic analysis of endothelial cells. All the above procedures were performed under sterile conditions. The thoracic aorta was dissected from the beginning of the aortic arch to the diaphragm and fixed in 10% neutral formalin for 24 h for the quantification of intimal plaque area.

### Plaque area quantification in the thoracic aorta

The percentage of plaque area in the thoracic aorta was determined according to a previous study [18]. The fixed thoracic aorta was dissected free of adhering fat and connective tissue and cut open longitudinally. The aorta was first washed in distilled water and rinsed in 60% isopropanol for 2 min, and then stained with 0.5% isopropanol solution of oil red O (Sigma, USA O0625) for 15 min, differentiated in 60% isopropanol three times (5 min each time), and finally washed in distilled water. Thereafter, the aorta was splayed, pinned plat on a white plate, and photographed using a digital camera (Canon EOS550D, Japan). The plaque area stained with oil red O (red stained) and total aortic surface area were measured using Image J (National Institutes of Health, USA). The extent of AS was assess by the rate of plaque area and the total intimal area (PA/IA).

### Vascular ring relaxation assay

The left common carotid artery was carefully dissected free of adhering fat and connective tissue and cut into four rings of 4 mm in length. Two rings with endothelial integrity were used for endothelium-dependent tension recording, and the other two endothelium-denuded rings were subjected to endothelium destruction by gently rubbing the intima with a toothpick for endothelium-independent tension recording. All the rings were carefully suspended between the bases of two triangular-shaped wires and placed into baths filled with oxygenated [95% oxygen (O_2_), 5% carbon dioxide (CO_2_)] modified Krebs solution (37 °C, pH 7.4), the Krebs solution contains the following composition (mM): sodium chloride (NaCl) 118.3, kalium chloratum (KCl) 4.7, potassium dihydrogen phosphate (KH_2_PO_4_) 1.2, magnesium sulphate (MgSO_4_) 1.2, sodium bicarbonate (NaHCO_3_) 24.9, calcium chloride (CaCl_2_) 2.5, Glucose 11.1. One end of each wire was connected to a force transducer, and the contractive signals passing through the transducer were recorded using the Powerlab system (AD instruments, USA). All the rings were then equilibrated at a resting tension of 1.5 g for at least 2 h. In all the procedures, care was taken to avoid any mechanical damage to the vascular rings. The bathing solution was exchanged with fresh Krebs solution every 15 min.

For endothelium-dependent tension recording, the intact rings were precontracted with phenylephrine (1 µM) and then relaxed by acetylcholine with a series of cumulative concentrations (1 × 10^− 9^, 3 × 10^− 9^, 1 × 10^− 8^, 3 × 10^− 8^, 1 × 10^− 7^, 3 × 10^− 7^, 1 × 10^− 6^, 3 × 10^− 6^, and 1 × 10^− 5^ M). For endothelium-independent tension recording, the endothelium-denuded rings were precontracted with phenylephrine (1 µM), and then relaxed by sodium nitroprusside with a series of cumulative concentration (1 × 10^− 9^, 3 × 10^− 9^, 1 × 10^− 8^, 3 × 10^− 8^, 1 × 10^− 7^, 3 × 10^− 7^, 1 × 10^− 6^, 3 × 10^− 6^, and 1 × 10^− 5^ M). For both endothelium-dependent and -independent tension recording, the rings exhibiting < 1 g tension when precontracted with phenylephrine (1 µM) were considered to be severely damaged and were discarded from the experiments. Relaxation of the rings was expressed as the percent reduction from the amount of phenylephrine-induced tension. Then cumulative concentration-response curves to acetylcholine or sodium nitroprusside were obtained.

### Aortic endothelial cell isolation and identification

The abdominal aorta was separated as described in the “Tissue Preparation” section and then put into sterile 4 °C RPMI 1640 medium (Gibco, USA) before being dissected free of adhering fat and connective tissue, cut open longitudinally, and washed with phosphate buffered saline (PBS) three times. Endothelial cells were isolated using modified methods described by a previous study [19]. The aorta intima was put downward and digested with 3.5 ml 0.2% collagenase (Sigma) at 37 °C for 15 min. Then 3.5 ml endothelial growth medium-2 (Lonza, Switzerland) supplemented with 10% fetal bovine serum (Gibco) was added to terminate the digestion. The solution was then transferred to a centrifuge tube, and cells were obtained after centrifugation [1000 revolutions per minute (rpm), 10 min]. The cells were seeded in a 24-well culture dish precoated with 0.1% gelatin and cultured with endothelial growth medium-2 supplemented with 10% fetal bovine serum.

The cultured cells were identified as endothelial cells based on their characteristic cobblestone morphology and positive staining for endothelial marker CD31. After 7 days, cultured cells were observed under an inverted fluorescent microscope (Leica, Wetzlar, Germany DMI4000B). For CD31 staining, cells with 80% confluency were fixed with 2% paraformaldehyde for 15 min, washed with PBS-tween (PBS-T), and incubated with 5% bovine serum albumin for 1 h to block nonspecific binding. The cells were then incubated with anti-CD31 antibody (clone JC/70A, Abcam, USA; dilution 1:10) for 20 h at 4 °C. After three washes with PBS-T, the cells were incubated with goat anti-mouse lgG complex (code ab96879, Abcam; dilution 1:20) for 55 min. Nucleus counterstaining was performed for 5 min using 4′,6-diamidino-2-phenylindole (DAPI) (5 µg/ml). The cells were then washed with PBS-T and mounted using 50% glycerin. Finally, the cells were viewed and photographed with the inverted fluorescent microscope. Cells demonstrating positive staining of CD31 in the cell membrane were identified as endothelial cells.

### Aortic endothelial cell apoptosis assay

The apoptosis of isolated endothelial cells of the abdominal aorta were evaluated. Cell apoptosis was assessed using an Annexin V-fluoresceine isothiocyanate (FITC) apoptosis detection kit (KeyGEN Biotech, China) according to the manufacturer’s instructions. Cells were washed twice with PBS and suspended in 0.5 ml binding buffer. Then the cell suspension was filtered to avoid possible impurity with larger mass. Subsequently, 5 µL of annexin V-FITC and 5 µL of propidium iodide (PI) were mixed with the cell suspension. After incubation for 15 min, mixtures were analyzed by flow cytometry (BD FACSCantoII, USA) within 30 min. Annexin V-negative and PI-negative cells were regarded as live cells, annexin V-positive and PI-negative cells were regarded as early apoptotic cells, while annexin V-positive and PI-positive cells were regarded as late apoptotic or secondary necrotic cells [20].

### Statistical analysis

This study was designed as three sub studies. The first sub study was designed to compare the normal and high-cholesterol groups and determine the effect of a hypercholesterolemic diet on AS. The second sub study was designed to compare the effects of different intensities of RTLI on AS and determine the minimum effective intensity of RTLI. The third sub study was designed to explore the mechanisms of the minimum effective intensity of RTLI on AS.

Data are presented as mean values ± standard deviation (SD) or median (interquartile range, IQR) as appropriate. Repeated values were analyzed with the analysis of variance (ANOVA) for repeated measures. The normal (N) and high-cholesterol (H) groups were compared with Student’s t-test (normal distribution) or Mann–Whitney U test (non-normal distribution). In the other sub studies, multiple comparisons for continuous normal distributed variables with homogeneity variance were done with one-way ANOVA followed by the least significant difference test for the differences between two groups. Normally distributed variables with heterogeneity variance or non-normal distributed variables were analyzed using compared using Kruskal Wallis test, resorting to a Mann–Whitney U test for comparisons between two groups. All analyses were performed using SPSS 16.0 statistical software package (SPSS Inc., Chicago, IL, USA). *P <* 0.05 was considered to indicate significance.

## Results

### General information

**Fig. 2:**
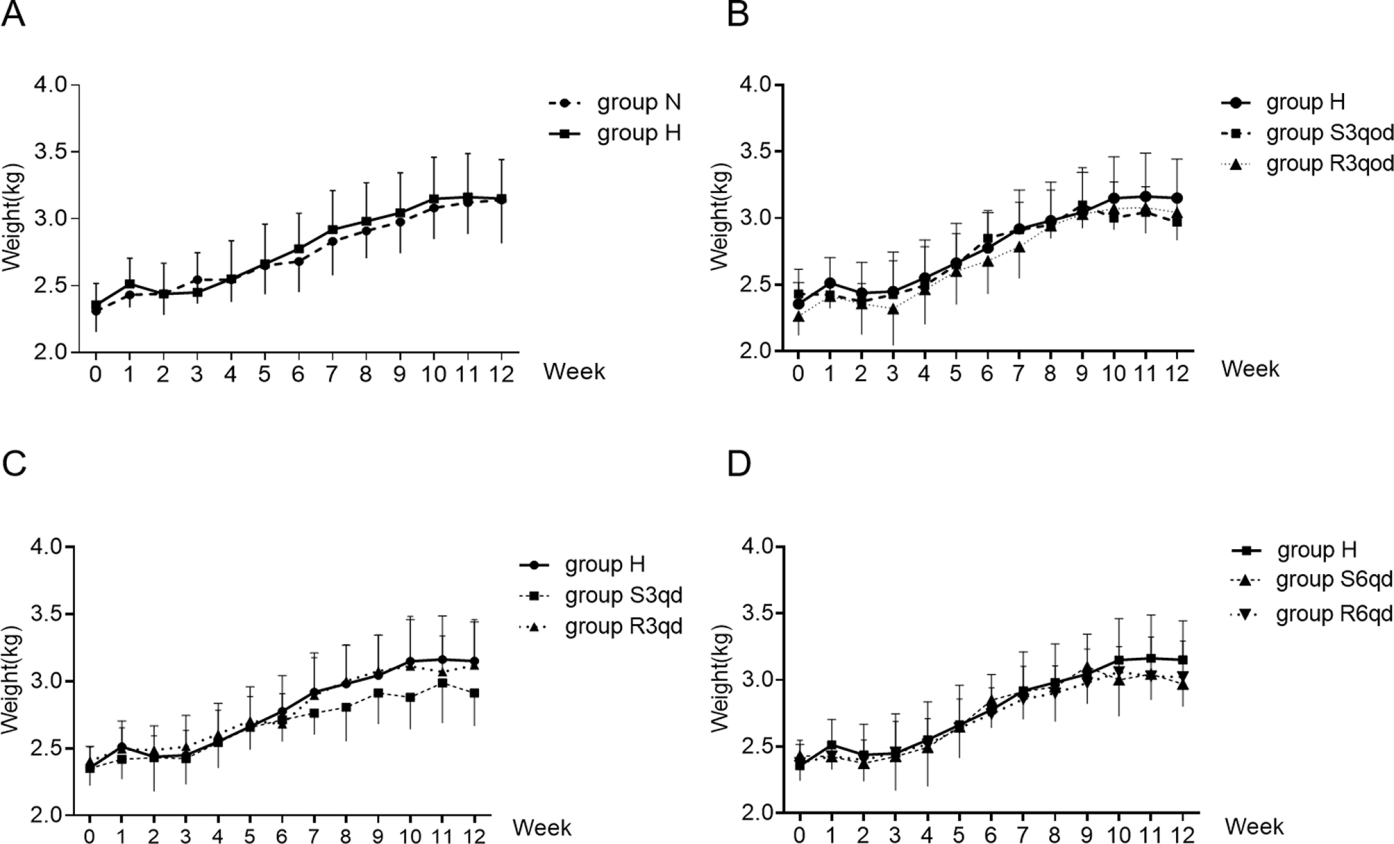
Effects of different intensities of RTLI on body weight gain of rabbits. (**A**) The difference of weight body gain in groups N and H was compared. (**B–D**) The comparison of weight body gain among groups H, S3qod, and R3qod (**B**), among groups H, S3qd, and R3qd (**C**), and among groups H, S6qd, and R6qd (**D**). Data are presented as mean ± SD; *n* = 10, 9, 8, 7, 8, 8, 8, and 9 in groups N, H, S3qod, R3qod, S3qd, R3qd, S6qd, and R6qd, respectively. RTLI, regular transient limb ischemia

The average amount of daily food intake per animal did not differ among the eight groups (all *P >* 0.05). No significant differences in weight gain were found among the eight groups over the 12-week period (all *P >* 0.05, Fig. 2A-D). A total of 13 rabbits were dropped from the study because of death, serious disease accompanied by a considerable reduction in food intake and serious infection, which was treated with antibiotics. A sharp reduction in food intake would inhibit the formation of AS, and serious infection accelerated the development of AS [19], which these influencing factors could interfere with the final results. The number of dropped animals in each group was as follows: One, two, three, two, two, two, and one dropouts in groups H, S3qod, R3qod, S3qd, R3qd, S6qd, and R6qd, respectively. Hence, a total of 10, 9, 8, 7, 8, 8, 8, and 9 rabbits finished this study in N, H, S3qod, R3qod, S3qd, R3qd, S6qd, and R6qd groups, respectively. There were no differences in the rate of death or severe illness among the eight groups (*P =* 0.821).

### Comparison of serum lipid

We detected the changes of serum lipid (HDL-C, LDL-C and TC) at the week 0, Week 6 and week 12. At week 0, the serum HDL-C, LDL-C, and TC level were no significant difference among eight groups (all *P >* 0.05). At week 6 and week 12, HDL-C, LDL-C, and TC levels in groups treated with high cholesterol were higher than those in group N (*P <* 0.05). At different time points, the HDL-C, LDL-C, and TC levels in R3qod group showed no significant difference compared to those in both groups H and S3qod (all *P >* 0.05); the HDL-C, LDL-C, and TC levels in R3qd group showed no significant difference compared to those in both groups H and S3qd (all *P >* 0.05); the HDL-C, LDL-C, and TC levels in S6qd group showed no significant difference compared to those in both groups H and S6qd (all *P >* 0.05) (Tables 1, 2 and 3).

**Table 1:** Serum HDL level in rabbits of each groups at different time points

Group	n	Week 0	Week 6	Week 12
N	10	0.82 ± 0.20	0.74 ± 0.22	0.54 ± 0.23
H	9	0.72 ± 0.11	1.67 ± 0.58*	2.68 ± 0.61*
S3qod	8	0.74 ± 0.23	1.59 ± 0.66*	2.56 ± 0.43*
R3qod	7	0.72 ± 0.24	1.84 ± 0.47*	2.67 ± 0.62*
S3qd	8	0.85 ± 0.20	2.04 ± 0.22*	2.86 ± 0.71*
R3qd	8	0.69 ± 0.11	1.71 ± 0.57*	2.51 ± 0.43*
S6qd	8	0.80 ± 0.19	2.05 ± 0.44*	2.90 ± 0.46*
R6qd	9	0.81 ± 0.27	1.87 ± 0.26*	2.37 ± 0.93*

All values are presented as mean ± SD (mmol/L). Week 0, before the experiment; Week 6, end of the sixth week; Week 12, end of the twelfth week; HDL, high-density lipoprotein cholesterol. *P < 0.05, compared to group N at the same time point. There was no significant difference in HDL level among groups H, S3qod, and R3qod; among groups H, S3qd, and R3qd; and among groups H, S6qd, and R6qd (*P >* 0.05)

**Table 2:** Serum LDL level in rabbits of each groups at different time points

Group	n	Week 0	Week 6	Week 12
N	10	0.78 ± 0.47	0.45 ± 0.19	0.49 ± 0.49
H	9	0.64 ± 0.24	10.89 ± 9.24*	18.95 ± 6.85*
S3qod	8	1.11 ± 1.08	9.68 ± 5.33*	19.82 ± 5.06*
R3qod	7	0.73 ± 0.36	9.22 ± 4.10*	17.67 ± 5.89*
S3qd	8	0.60 ± 0.11	10.49 ± 3.29*	22.79 ± 4.21*
R3qd	8	0.58 ± 0.17	9.27 ± 5.03*	18.29 ± 5.69*
S6qd	8	0.60 ± 0.17	12.60 ± 8.20*	19.31 ± 5.32*
R6qd	9	0.78 ± 0.54	9.93 ± 3.62*	16.97 ± 5.35*

All values are presented as mean ± SD (mmol/L). Week 0, before the experiment; Week 6, end of the sixth week; Week 12, end of the twelfth week; LDL, low-density lipoprotein cholesterol. *P < 0.05, compared to group N at the same time point. There was no significant difference in LDL level among groups H, S3qod, and R3qod; among groups H, S3qd, and R3qd; and among groups H, S6qd, and R6qd (*P >* 0.05)

**Table 3:** Serum TC level in rabbits of each groups at different time points

Group	n	Week 0	Week 6	Week 12
N	10	1.87 ± 0.66	1.22 ± 0.38	1.20 ± 0.74
H	9	1.56 ± 0.28	19.86 ± 17.94*	33.27 ± 11.85*
S3qod	8	2.16 ± 1.41	20.27 ± 14.99*	36.86 ± 10.30*
R3qod	7	1.66 ± 0.49	19.12 ± 9.65*	34.23 ± 6.87*
S3qd	8	1.66 ± 0.30	19.92 ± 9.45*	41.25 ± 8.24*
R3qd	8	1.49 ± 0.22	19.05 ± 15.41*	35.07 ± 10.01*
S6qd	8	1.64 ± 0.32	23.90 ± 16.13*	34.37 ± 8.96*
R6qd	9	1.94 ± 0.98	21.30 ± 12.90*	32.58 ± 10.75*

All values are presented as mean ± SD (mmol/L). Week 0, before the experiment; Week 6, end of the sixth week; Week 12, end of the twelfth week; TC, total cholesterol. *P < 0.05, compared to group N at the same test point. There was no significant difference in TC level among groups H, S3qod, and R3qod; among groups H, S3qd, and R3qd; and among groups H, S6qd, and R6qd (*P >* 0.05)

### Plaque area quantification in the thoracic aorta

**Fig. 3:**
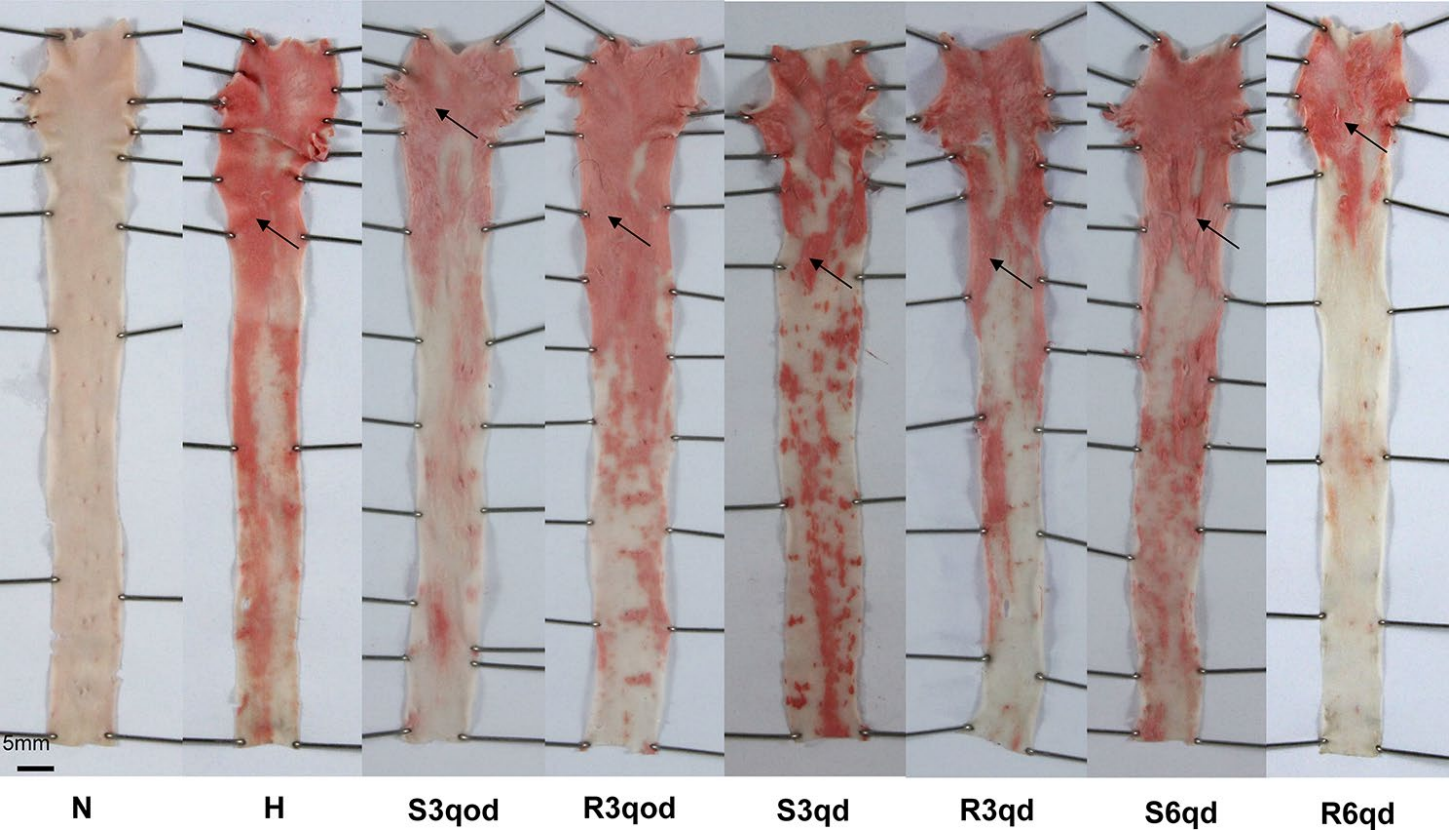
Representative images of the thoracic aorta by oil red O staining in each groups. The plaque lesions are showed as red. No lesion was seen in group N. Obvious lesions were seen in the other groups. “→” indicates the plaque lesions

**Fig. 4:**
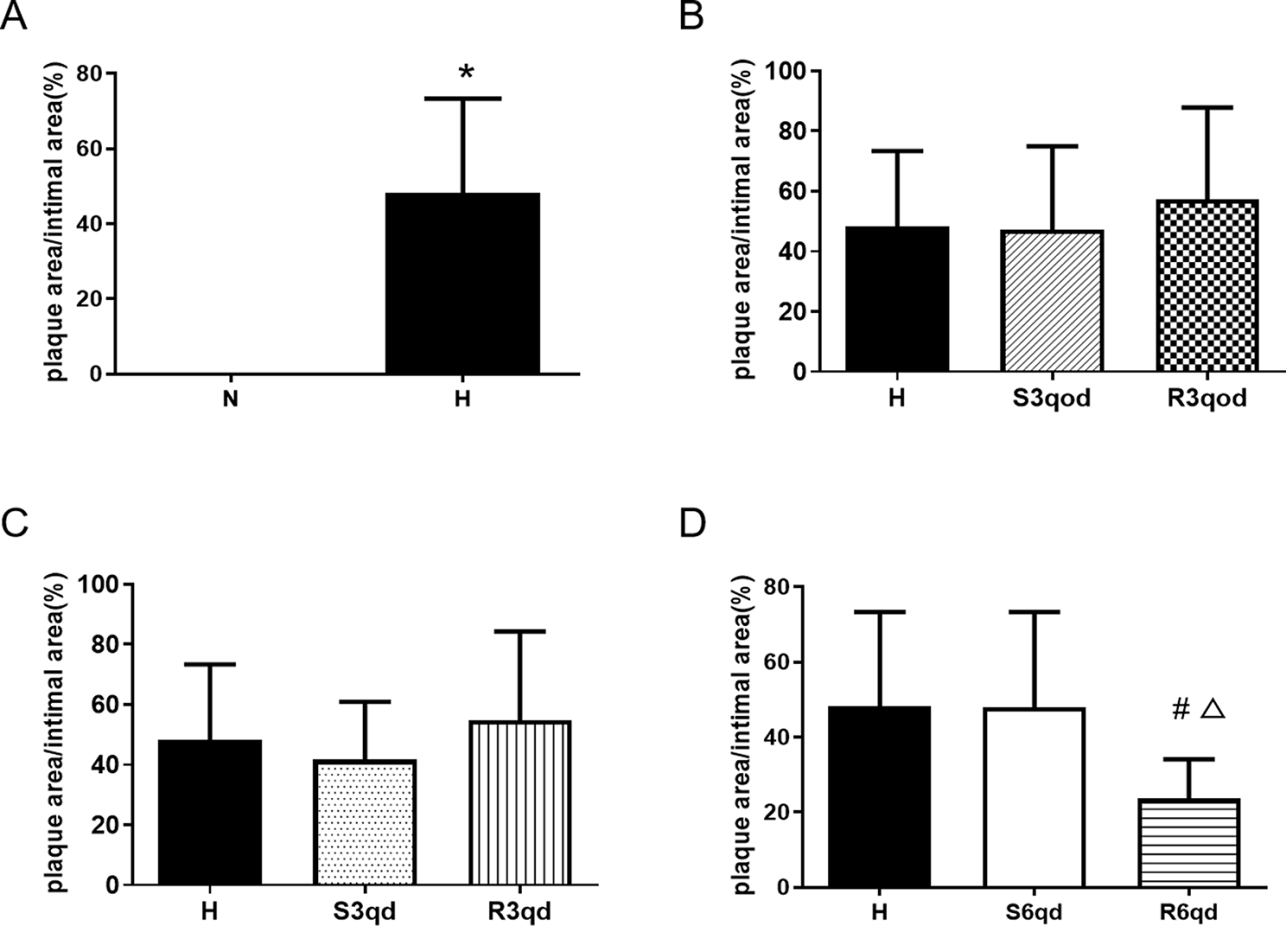
Effects of different intensities of RTLI on the percentage of plaque area in the aortic intimal area (PA/IA). (**A**) Comparison of PA/IA between groups N and H. *P < 0.05. (**B**) Comparison of PA/IA among groups H, S3qod and R3qod. (**C**) Comparison of PA/IA among groups H, S3qd, and R3qd. (**D**) Comparison of PA/IA among groups H, S6qd, and R6qd. *P < 0.05 compared with group H; Δ*P <* 0.05 compared with group S6qd. Data are presented as mean ± SD, *n* = 10, 9, 8, 7, 8, 8, 8, and 9 in groups N, H, S3qod, R3qod, S3qd, R3qd, S6qd, and R6qd, respectively

The aortic intima in the N group was smooth and had no red-stained lipid-rich lesions. In contrast, there were red-stained lipid-rich lesions in the intima in the other groups fed the hypercholesterolemic diet. The lesions were flat or slightly protruding into the lumen (Fig. 3). There was significant differences in the PA/IA between groups N (0 ± 0%) and H (48.17 ± 25.19%) (*P <* 0.001, Fig. 4A). The PA/IA in group R6qd (23.65 ± 10.43%) was significantly lower than that in groups H (*P =* 0.023) and S6qd (48.01 ± 25.34%) (*P =* 0.028, Fig. 4D). However, the PA/IA in R3qod group (57.19 ± 30.62%) showed no significant differences compared to that in both groups H and S3qod (47.13 ± 27.87%) (*P =* 0.748); the PA/IA in R3qd group (54.83 ± 29.48%) showed no significant differences compared to that in both groups H and S3qd (41.71 ± 19.22%) (*P =* 0.584) (Fig. 4B-C).

### Endothelium-dependent and -independent tension of vascular ring

**Fig. 5:**
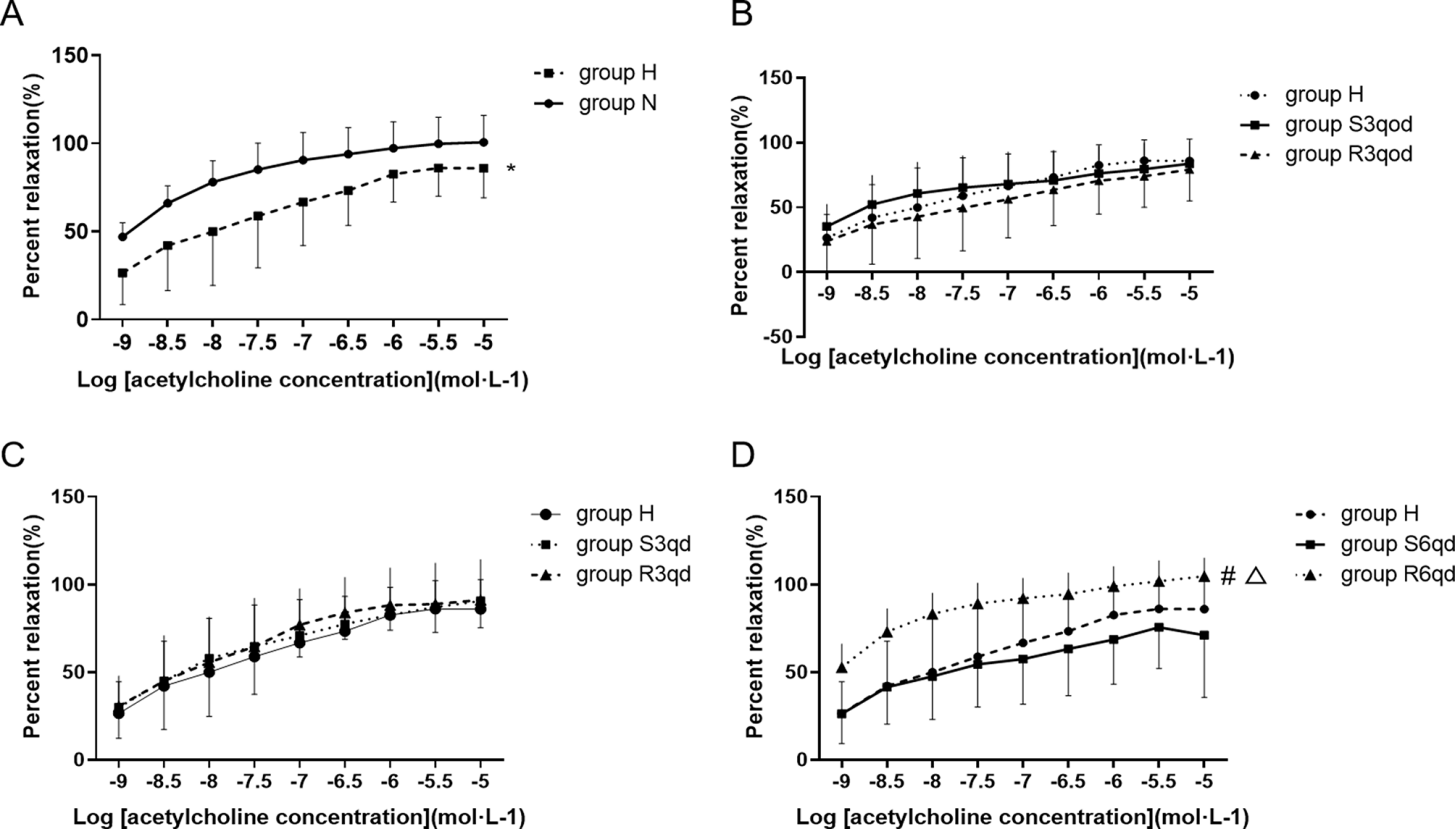
Effects of different intensities of RTLI on the endothelium-dependent tension of vascular ring. (**A**) Comparison of relaxation responses to acetylcholine between groups N and H. *P < 0.05. (**B**) Comparison of the relaxation response to acetylcholine among groups H, S3qod, and R3qod. (**C**) Comparison of the relaxation response to acetylcholine among groups H, S3qd, and R3qd. (**D**) Comparison of relaxation response to acetylcholine among groups H, S6qd, and R6qd. *P < 0.05 compared with group H; Δ*P <* 0.05 compared with group S6qd. Data are presented as the mean ± SD, *n* = 10, 9, 7, 6, 8, 7, 6, and 9 in groups N, H, S3qod, R3qod, S3qd, R3qd, S6qd, and R6qd, respectively

Acetylcholine (1 × 10^− 9^–1 × 10^− 5^M) produced concentration-dependent relaxation in phenylephrine-precontracted arterial rings with endothelium in all groups. Compared with that in group N, the endothelium-dependent relaxation was impaired in group H (*P =* 0.020, Fig. 5A). The endothelium-dependent relaxation in group R6qd was considerably higher than that in groups H (*P =* 0.012) and S6qd (*P =* 0.008, Fig. 5D). However, no differences in endothelium-dependent relaxation were observed among groups H, S3qod, and R3qod (*P =* 0.730, Fig. 5B); and among groups H, S3qd, and R3qd (*P =* 0.948, Fig. 5C).

**Fig. 6:**
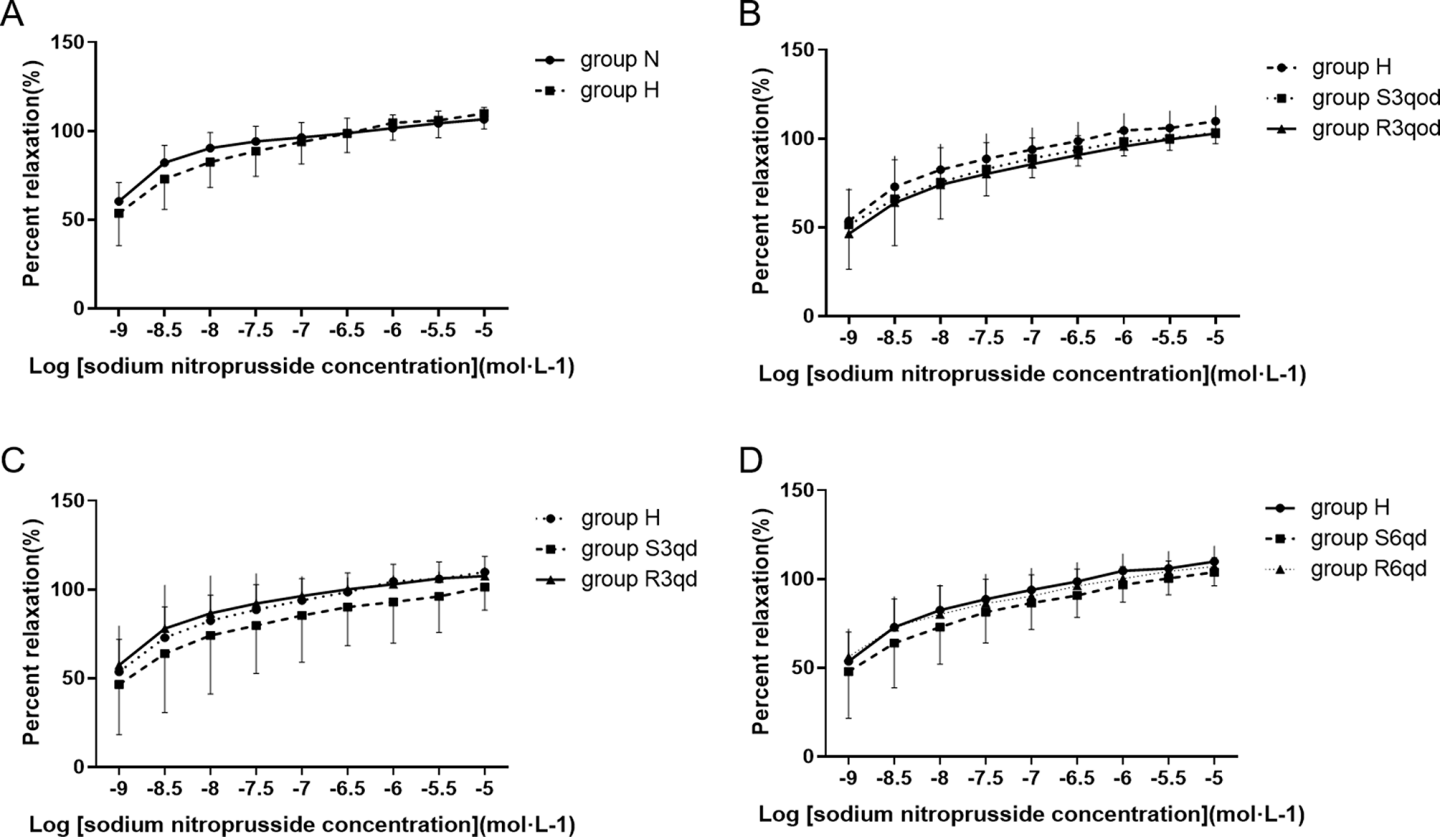
Effects of different intensities of RTLI on the Endothelium-independent tension of vascular ring. (**A**) Comparison of relaxation responses to sodium nitroprusside between groups N and H. (**B**) Comparison of the relaxation response to sodium nitroprusside among groups H, S3qod, and R3qod. (**C**) Comparison of the relaxation response to sodium nitroprusside among groups H, S3qd, and R3qd. (**D**) Comparison of relaxation response to sodium nitroprusside among groups H, S6qd, and R6qd. Data are presented as the mean ± SD, *n* = 10, 9, 7, 6, 8, 7, 7, and 9 in groups N, H, S3qod, R3qod, S3qd, R3qd, S6qd, and R6qd, respectively

Regarding endothelium-independent relaxation response, sodium nitroprusside (1 × 10^− 9^–1 × 10^− 5^ M) also produced concentration-dependent relaxation in phenylephrine-precontracted endothelium-denuded arterial rings in all groups. However, the total endothelium-independent relaxation responses were not significantly different among the eight groups (all *P >* 0.05, Fig. 6A-D).

### Aortic endothelial cell isolation and identification

**Fig. 7:**
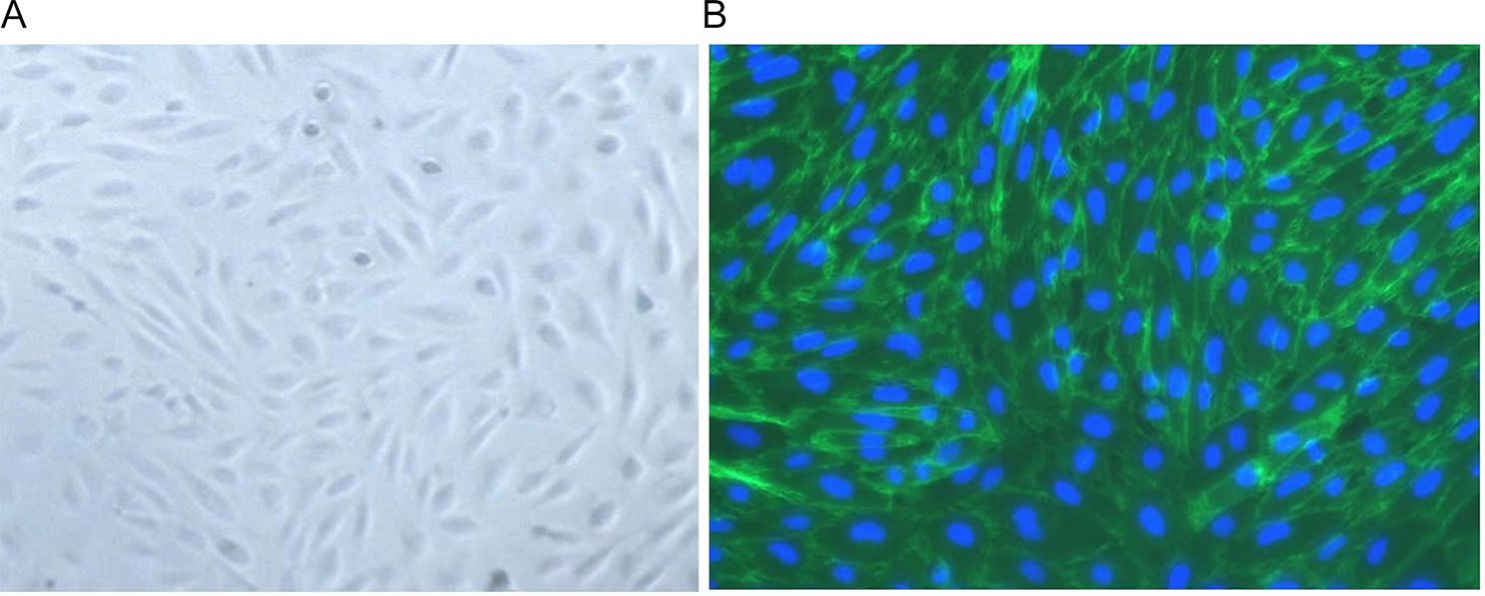
Identification of aortic endothelial cells. (**A**) Typical micrograph of isolated aortic endothelial cells was observed under an inverted microscope (×100). (**B**) Representative image of endothelial cells by CD31 immunofluorescence staining was obtained under an inverted fluorescence microscope (×200). Cells nucleus are stained by DAPI (blue). CD31 positive cells exhibit green fluorescence. Above 95% of the cells are expressed CD31 positive

The cultured endothelial cells from the abdominal aorta adhered after 2 days and proliferated rapidly after 4 days. After 7 days of cultivation, cells developed into spindle shapes and exhibited the characteristic cobblestone-like appearance (Fig. 7A). More than 95% of these cultured cells expressed positive reactivity to CD31 antibody (Fig. 7B).

### Apoptosis ratio of aortic endothelial cells

**Fig. 8:**
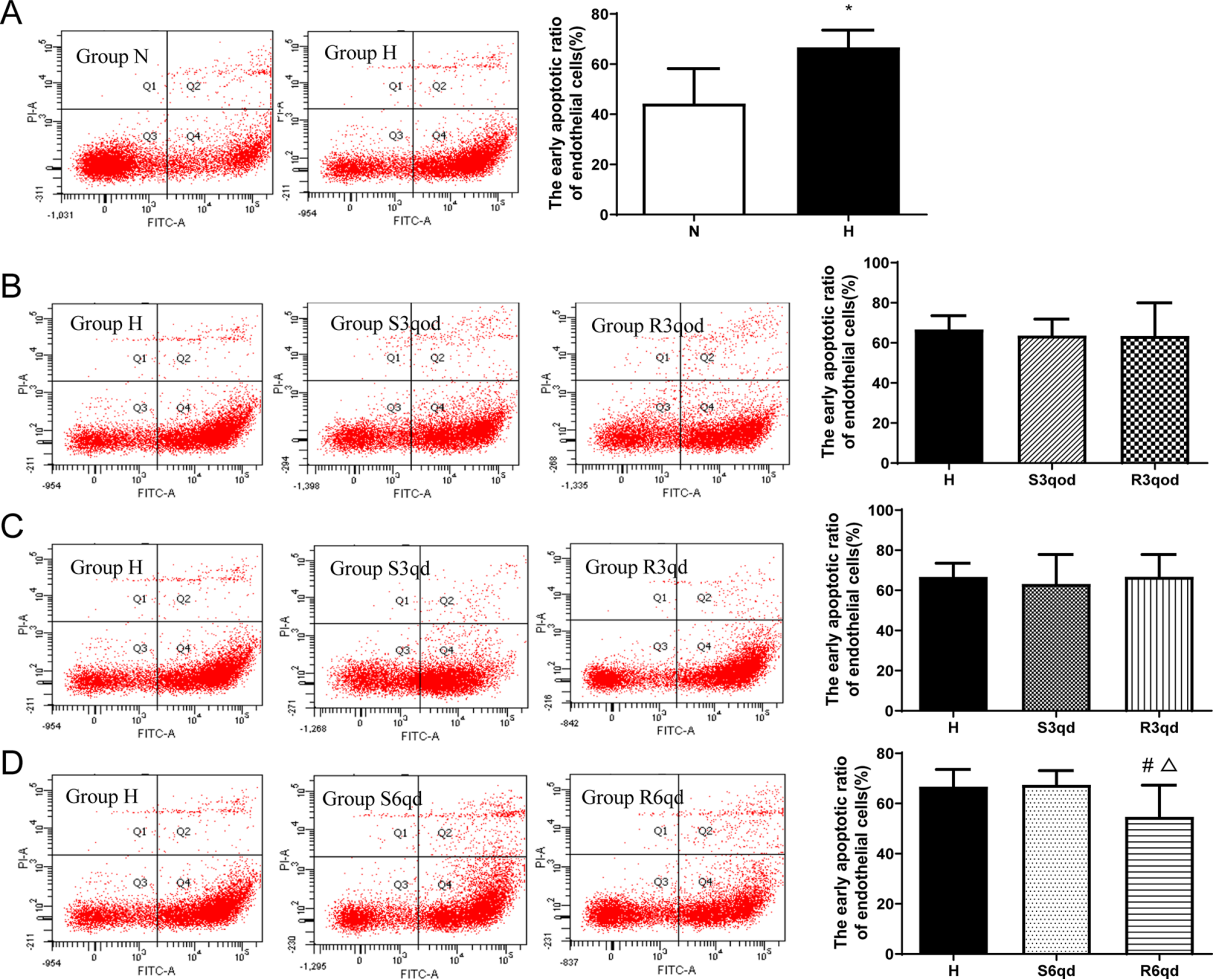
Effects of different intensities of RTLI on the early apoptotic ratio of the endothelial cells. The cell early apoptotic ratio was detected by flow cytometry. (**A**) Comparison of differences in the early apoptotic ratio of endothelial cells between groups N and H. *P < 0.05. (**B**) Comparison of differences in the early apoptotic ratio of endothelial cells among groups H, S3qod and R3qod. (**C**) Comparison of differences in the early apoptotic ratio of endothelial cells among groups H, S3qd and R3qd. (**D**) Comparison of differences in the early apoptotic ratio of the endothelial cells among groups H, S6qd and R6qd. *P < 0.05 compared with group H; Δ*P <* 0.05 compared with group S6qd. Data are presented as the mean ± SD, *n* = 10, 9, 7, 7, 8, 7, 8, and 9 in groups N, H, S3qod, R3qod, S3qd, R3qd, S6qd, and R6qd, respectively

We detected the apoptosis ratio of endothelial Cells by flow cytometry. Compared with group N (44.33 ± 13.88%), group H (66.73 ± 6.85%) had a higher early apoptotic ratio (*P <* 0.001, Fig. 8A). As shown in Fig. 8D, the early apoptotic ratio in group R6qd (54.70 ± 12.58%) was significantly reduced compared with that in either group H (66.73 ± 6.85%, *P =* 0.009) or group S6qd (67.45 ± 5.66%, *P =* 0.008). Furthermore, the early apoptotic ratio in R3qod group showed no significant differences compared to that in groups H and S3qod (all *P >* 0.05); the early apoptotic ratio in R3qd group showed no significant differences compared to that groups H and S3qd (all *P >* 0.05) (Fig. 8B-C).

## Discussion

Our study showed that six cycles of RTLI daily could reduce AS plaque area proportion, ameliorate endothelial relaxation dysfunction, and inhibit endothelial apoptosis in hypercholesterolemic rabbits. But the lower intensities of RTLI, including three cycles of RTLI once every other day (R3qod) and once daily (R3qd), showed no preventive effects against AS progression. These results indicated that six cycles of RTLI once daily was the optimal effective intensity of RTLI for anti-atherogenic effects, and the decrease in endothelial relaxation dysfunction and apoptosis might be important targets in such RTLI-induced anti-atherogenic effects.

In a previous study, it was reported that six cycles of RTLI once daily for 12 weeks could reduce the aortic plaque area in hypercholesterolemic rabbits [12]. In the present study, we showed that six cycles of RTLI once daily had the same effect, indicating the considerable reliability and repeatability of this study protocol. These findings suggest that RTLI might be a novel, effective, and non-invasive intervention to manage AS. However, whether the lower intensities of RTLI (less than six cycles once daily) had similar preventive effects were undetermined.

Previous studies showed that different intensities of TLI had different effects on ischemia and reperfusion injury [21, 22]. Repeated TLI consisting of three cycles of 5 min ischemia/5 min reperfusion daily for 3 days could reduce post-myocardial ischemia/reperfusion injury in diabetic mice [21]. Johnsen et al. observed similar cardiac protection with a single administration of four, six, or eight cycles of 5 min TLI, but not with two cycles in an isolated rat heart model using Langendorff apparatus [22]. In the present study, we found that six cycles of RTLI once daily could reduce the aortic intimal plaque formation. However, the lower intensities, including three cycles of RTLI once every other day and three cycles of RTLI once daily, could not reduce the plaque area, which indicated that these lower intensity interventions were not strong enough or the duration was insufficient to have a preventive effect against AS plaque. To the best of our knowledge, there has been no other study on the effects of different intensities of long-term RTLI on AS prevention. From our results, we consider that six cycles of RTLI once daily was the optimal effective intensity of RTLI for AS prevention.

Endothelial dysfunction is characterized by reduced nitric oxide bioavailability, which then leads to impairment of endothelium-dependent vasodilation which is considered as the functional characteristic of endothelial dysfunction [23]. Besides endothelium-dependent vasodilation, endothelium-independent vasodilation is another form of vascular relaxation. These two forms of vascular relaxation can be assessed in vivo and in vitro. Previous literature showed that vascular relaxation in vitro is a common and direct method to assess vascular function in animals [24]. Therefore, the relaxation response of isolated common carotid artery was used to evaluate the vascular function in this study. Vascular relaxation induced by acetylcholine is dependent on the presence of endothelial cells and vascular relaxation induced by sodium nitroprusside is endothelium-independent [25]. Zhang et al. showed that endothelium-dependent relaxation to acetylcholine is impaired by hypercholesterolemia but not endothelium-independent relaxation to sodium nitroprusside [26]. Our study indicated the consistent results. We found that six cycles of RTLI once daily for a 12 week duration could improve endothelium-dependent relaxation but not the endothelium-independent relaxation. These results showed that RTLI was able to protect endothelial cells and then reduce endothelial dysfunction induced by hypercholesterolemia. Liang et al. [15] and Kimura et al. [10] also showed the protective effects of TLI on endothelial cells. Therefore, we confirmed our hypothesis that the endothelial cells might be a key target of the anti-atherosclerotic effects of RTLI.

Endothelial apoptosis occurs in an early stage in the pathogenesis of AS [27], and even contributes to plaque erosion and acute coronary syndromes [28]. Increased apoptosis of endothelial cells has been observed in atheromatous lesions. Additionally, the expression of pro-apoptotic proteins, such as Factor Related Apoptosis (Fas) and B-cell lymphoma-2-Associated X (Bax), increased, while levels of anti-apoptotic factors decreased in endothelial cells overlying the lesions [29]. In contrast, over-apoptosis of endothelial cells would lead to a decrease in endothelial cells and thus endothelial dysfunction [30]. Therefore, anti-apoptotic therapy is considered an effective strategy to inhibit the development and progression of AS [31]. Previous studies have demonstrated that RTLI is capable of protecting endothelial cells and reducing endothelial dysfunction. In the present study, we found that six cycles of RTLI daily inhibit endothelial apoptosis, while the milder RTLI (R3qod and R3qd) did not have this effect.

Both in vivo and in vitro studies have showed that ischemic preconditioning could protect endothelial cells from apoptosis caused by ischemia and reperfusion injury. Taha et al. reported that ischemic preconditioning induced by 5 min of ischemia followed by 10 min of reperfusion could decrease the gene expression of pro-apoptotic markers and increase the expression of anti-apoptotic genes in the endothelial cells after the subsequent ischemia and reperfusion [32]. Bellis et al. demonstrated that ischemic preconditioning decreased hypoxia-induced apoptosis in bovine aortic endothelial cells in vitro [33]. In our study, a decreased apoptotic ratio of endothelial cells was observed in the ischemia group, suggesting that RTLI might have anti-apoptotic effects against endothelial cells under hypercholesterolemic conditions.

Nonetheless, our study had some limitations. Firstly, our study lacked evidence confirming endothelial apoptosis in vivo. Furthermore, the underlying mechanisms accounting for RTLI-induced anti-apoptotic effects remain unknown. Further studies are needed to explore the above mechanisms. Some studies have indirectly tried to explain the anti-apoptotic effects mediated by ischemic preconditioning; such studies focused on protein kinase A (PKA)- and phosphatidylinositol-3-kinase (PI3K)-dependent activation of Akt [34], pro-apoptotic markers (such as Caspase 1 and Caspase 6), and anti-apoptotic genes [B-cell lymphoma-2 (Bcl-2) and neuronal apoptosis inhibitory protein 2 (Naip2)] [35]. However, RTLI is not ischemic preconditioning, so whether the same mechanisms would be shared, remains to be ascertained. Secondly, the mechanisms that account for the RTLI-induced anti-atherosclerotic effects are complex, and as our study solely focused on endothelial vasomotor function and apoptosis, detailed descriptions of the mechanisms were not included. Therefore, these mechanisms should be elucidated in future studies. Thirdly, the subjects in our study were New Zealand White rabbits only, so whether these findings could be translated to other animals or humans is unknown. Further investigations are required to define such beneficial effects of RTLI in other animals and, more importantly, humans.

## Conclusion

Taken together, our results indicated that six cycles of RTLI daily was the optimal effective intensity to prevent AS progression in rabbits. Six cycles of RTLI daily could greatly reduce the plaque area and ameliorate endothelial relaxation dysfunction in hypercholesterolemic rabbits, inhibit endothelial apoptosis.

## Data Availability

The data of this study can be obtained from the first author if reasonably requested.
